# DELPHI expert consensus-derived factors influencing the early use of monoclonal high-efficacy therapies in treatment-naïve patients with relapsing-remitting multiple sclerosis

**DOI:** 10.1007/s00415-026-14167-4

**Published:** 2026-10-22

**Authors:** Bertrand Bourre, Olivier Guerrier, Xavier Ayrignac, Laurent Suchet, Jérôme de Seze, Ines Masmoudi, Vito A. G. Ricigliano, Patrick Vermersch, Jonathan Ciron

**Affiliations:** 1 Department of Neurology and Inserm, CIC-CRB 1404, CHU Rouen, F-76000 Rouen, France; 2 Department of Neurology and Neurovascular Unit, Valence Hospital, Valence, France; 3 Département de Neurologie, CRC sclérose en plaques, CRMR LEUKOFRANCE & Centre d’investigation clinique U1411, CHU de Montpellier, INM, INSERM, Univ Montpellier, Montpellier, France; 4 Department of Neurology, European Hospital of Marseille, Marseille, France; 5 Department of Neurology and Clinical Investigation Center, Strasbourg University Hospital, Strasbourg, France; 6 Department of Neurology, Amiens University Hospital, Amiens, France; 7 Neurology Unit, GHNE Paris Saclay Hospital, Orsay, France; 8 UNIACT, Neurospin—INSERM UMR 1129, CEA, Université Paris-Saclay, Gif-sur-Yvette, France; 9 INSERM U1172 LilNCog, Lille University Hospital, FHU Precise, University of Lille, Lille, France; 10 Department of Neurology, CRC-SEP, Toulouse University Hospital, Toulouse, France; 11 INSERM UMR1291—CNRS UMR5051, INFINITY, University Toulouse III, Toulouse, France

**Keywords:** Multiple sclerosis, Relapsing-remitting multiple sclerosis, Monoclonal antibodies, Consensus statement, Delphi technique, France

## Abstract

**Objective:**

To gather expert consensus regarding initiation of monoclonal high-efficacy therapies (HET) amongst treatment-naïve relapsing-remitting multiple sclerosis (RRMS) patients in France.

**Methods:**

The study leveraged the Delphi method to gauge consensus amongst an expert panel of 31 neurologists. Eight clinical cases with demographic, clinical and imaging characteristics were presented, prompting the experts to respond if they would recommend monoclonal HET. Additionally, potentially influential parameters were changed one at a time to assess whether the experts would recommend monoclonal HET. After a first round, an e-workshop was conducted to discuss non-consensual statements, which were re-submitted for rating (Round 2). Characteristics influencing monoclonal HET initiation were evaluated.

**Results:**

31/31 experts responded to both rounds. Consensus for or against monoclonal HET was reached for 89/108 (82.4%) statements. Factors that systematically led to monoclonal HET initiation were high brain lesion load (>20 lesions), ≥2 spinal cord lesions, ≥3 gadolinium-enhancing lesions and ≥3 infratentorial lesions. A single spinal cord lesion, in combination with other factors (which, in isolation, often but not always led to monoclonal HET), systematically led to monoclonal HET recommendation.

**Conclusion:**

This study suggests that the panel of French neurologists favoured monoclonal HET initiation amongst treatment-naïve RRMS patients with high radiological disease activity, whereas clinical characteristics played a complementary role.

**Supplementary Information:**

The online version contains supplementary material available at https://doi.org/10.1007/s00415-026-14167-4.

## Introduction

Multiple sclerosis (MS) imposes a substantial burden amongst affected patients. Given the relatively early age of onset and the incapacitating clinical consequences of neurodegeneration, MS represents a major healthcare and public health challenge [1]. The relapsing-remitting MS (RRMS) form is experienced by most patients and is characterised by intermittent flare-ups of neuroinflammation and demyelination, and the consequent neurodegeneration that leads to accrued disability [2, 3].

Disease-modifying therapies (DMT) are treatment options that aim to change the long-term course of the disease, reducing the relapse rate and the risk of new lesions seen by magnetic resonance imaging (MRI), thus slowing the accumulation of disability. There are different types of DMT, and they can be classified according to their relative efficacy in controlling disease: moderate-efficacy therapies (MET) and high-efficacy therapies (HET). The former group includes interferon‑β, glatiramer acetate, teriflunomide, and fumarates. Meanwhile, the latter higher-efficacy agents include monoclonal antibodies, such as anti-CD20 and anti-α4-integrin antibodies, as well as cladribine and S1P receptor modulators [1, 3].

A systematic review of clinical trials by Cagol and colleagues showcased the efficacy of HET in preventing brain volume loss, MRI lesion activity and disability progression [4]. The effect sizes estimated for HET compared to placebo were considerably stronger than those for MET against placebo. Furthermore, within the therapies classified as HET, several studies suggest that monoclonal HET is likely to be superior in controlling disease progression [4–6], and will, therefore, be the focus of the present study. Notwithstanding, the efficacy of these therapies is often weighed against potential safety concerns, such as progressive multifocal leukoencephalopathy or hypogammaglobulinemia and consequent infections [3].

Consequently, there are two broad paradigms for the treatment approach in patients recently diagnosed with RRMS: an escalation strategy and an early intensive approach. For decades, the standard and most frequent approach has been to start with MET and to only escalate to higher-efficacy alternatives if the inflammatory activity persists (as evidenced by new lesions on imaging, with or without relapses) [3]. This escalation approach can be considered conservative, as MET have a generally favourable safety profile. However, the counterargument is that the patient can be at higher risk of relapses and accruing irreversible disability whilst under MET [3, 7].

Over the past decade, a more ‘aggressive’ approach that implies an earlier start of HET is being increasingly applied [3]. There is real-world evidence yielding positive results in favour of an early start of HET vs an escalation approach [7–11]. Consequently, the early intensive treatment paradigm has gained momentum [12] and the use of HET (notably monoclonal HET) in treatment-naïve patients has increased over time. A recent study using data from the French National Health Data System (*Système National des Données de Santé*—SNDS) described the prescription of disease-modifying therapies amongst patients newly diagnosed with MS in the French healthcare system from 2016-2023 [13]. Comparing the periods encompassing 2016–2018 and 2022–2023, the use of anti-CD20 and anti-α4-integrin antibodies as first-line therapies grew approximately by 19-fold (1.3% vs 24.4%) and 2-fold (3.9% vs 8.6%), respectively. Meanwhile, the use of S1P modulators and cladribine remained relatively stable, whereas a decline was observed in the use of MET as first-line agents. It is important to note that this significant increase in the use of newer anti-CD20 therapies can be largely explained by their marketing authorisation in France, which occurred between the two studied periods [14, 15].

Similarly, Didierjean and colleagues evaluated prescription patterns between 2007 and 2022 in the Alsace region of eastern France and found that the time from RRMS diagnosis to the first prescription of a HET became shorter over time [16]. A study amongst French neurologists enquired about their treatment approach for hypothetical clinical cases and found an inclination to start HET earlier in the course of disease [17].

It is recommended that the therapeutic approach be based on patients’ characteristics. There is strong consensus regarding the need to personalise therapy schemes to each individual to optimise for both efficacy and safety [18]. For example, a recent systematic review of clinical trials found that the therapeutic benefit of HET was greater amongst patients with higher relapse rates [19].

Despite the increasing use of HET at earlier disease stages, there is no clear consensus on which treatment-naïve patients should receive HET, particularly monoclonal HET, as first-line therapy. Additionally, the American Academy of Neurology highlighted in their practice guidelines recommendations (from 2018, reaffirmed in 2024) that evidence is still needed to determine if early initiation of HET is associated with improved clinical outcomes in the longer term [20]. There are currently two large ongoing randomised clinical trials aiming to evaluate the efficacy of early initiation of HET amongst treatment-naïve patients: the TREAT-MS [21] and DELIVER-MS [22] trials. Both trials are yet to produce results. The lack of trial evidence and limited treatment consensus result in substantial variability in clinical practice and uncertainty in decision-making.

Although recent consensus and vignette-based studies have addressed individualised treatment decisions in patients with RRMS [12, 17, 23], the place of monoclonal HET within the broader therapeutic strategy is still not fully determined and evidence examining the factors influencing its initiation in the French context remains limited. The present study seeks to contribute evidence in this area. In the light of this situation, the aim of the study was to explore expert consensus and identify key factors driving experts’ decision to initiate monoclonal HET amongst treatment-naïve RRMS patients in France.

## Methods

### Study design

The present study leverages the Delphi methodology [24, 25] to gather consensus amongst French neurologists, regarding the early use of monoclonal HET amongst treatment-naïve RRMS patients. For the present study, the relevant treatments include only monoclonal HET (i.e. anti-CD20 and anti-α4-integrin antibodies), as these agents have shown higher efficacy than other pharmacological classes, including S1P modulators and cladribine [4, 6, 26, 27].

**Fig. 1:**
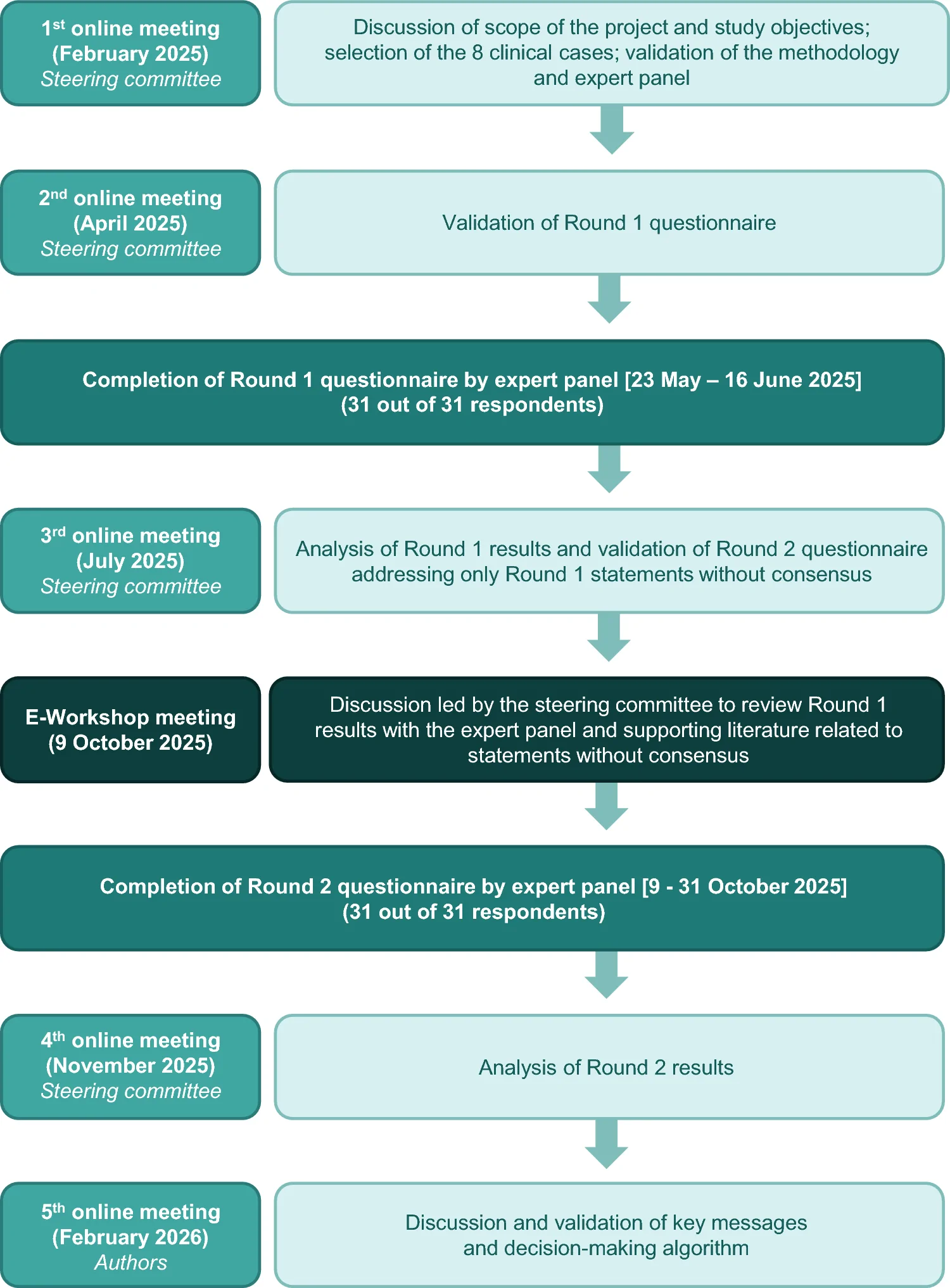
Schematic overview of the Delphi process conducted to explore expert consensus and identify key factors driving the initiation of monoclonal high-efficacy therapies amongst treatment-naïve patients diagnosed with relapsing-remitting multiple sclerosis, France

Seven of the authors (XA, BB, JC, OG, JDS, LS, PV), neurologists from France with an expertise in MS, will be hereafter referred to as the ‘steering committee’. The steering committee designed the study methodology and conducted the procedures explained below. Members of the steering committee did not participate in the voting process. An overview of the stages and timeframes of the study can be found in Fig. 1.

### Participants: the expert panel

Neurologists considered for participation in this study were identified by the steering committee, based on their expertise in the management of patients with MS and experience in the use of monoclonal HET. The selection process also aimed to ensure broad geographical coverage across France and representation of the different healthcare settings involved in MS care. From an initial list of 41 neurologists, 31 were ultimately selected and invited to participate in the study, hereafter collectively referred to as the ‘Expert Panel’. The invited experts worked across 28 locations in France and included 19 neurologists working in University Hospitals (*Centres Hospitaliers Universitaires, CHU*), 8 in General Hospitals (*Centres Hospitaliers generaux, CHG*) and 4 in private practices.

Two members of the expert panel (IM, VAR), who took part in the voting process, subsequently contributed to the final discussion and validation of the study findings and are therefore listed amongst the authors.

### Questionnaire structure

The questionnaire was developed by the steering committee through an iterative process. The questionnaire enquired about the therapeutic approach the members of the expert panel would follow when presented with a series of eight fictional clinical cases.

The cases included brief information regarding sociodemographic characteristics (sex, age, ethnicity), medical history (comorbidities), clinical presentation of the initial attack (severity of symptoms, motor impairment), and cognitive impairment and MRI findings at diagnosis (brain lesion load, number of infratentorial lesions, number of spinal cord lesions, and number of Gadolinium‑enhancing lesions). The brain lesion load was classified as low (<10 lesions), moderate (10-20 lesions) and high (>20 lesions). MRI scans of these categories can be found in Online Resource 1. A detailed description of the cases can be found in Table 1. The order in which the cases were presented to the participants was random. The participants had to respond if they would recommend initiating a monoclonal HET: “In this situation, would you recommend a monoclonal antibody HET?”. Participants were asked to respond to each scenario using a Likert-like scale ranging from 1 to 5, where 1 meant “Not at all in agreement with the proposed therapeutic approach” and 5 meant “In full agreement with the proposed therapeutic approach”. Scores of 4 and 5 were deemed to be in the “Indication” area (treatment advised), a score of 3 fell into the “Indecision” area and scores of 1 and 2 denoted a “No indication” area (treatment not advised).

**Table 1:** Overview of the eight clinical cases included in the Delphi study to explore expert consensus and identify key factors driving the initiation of monoclonal high-efficacy therapies amongst treatment-naive relapsing-remitting multiple sclerosis patients, France

Type of profile	Not severe	Intermediate			Severe			
Classification established based on Round 1 results	Clinical case 1	Clinical case 2	Clinical case 3	Clinical case 4	Clinical case 5	Clinical case 6	Clinical case 7	Clinical case 8
Case description								
Patient characteristics	25-year-old Caucasian woman	32-year-old Maghrebi man	27-year-old Caucasian man	35-year-old Afro-Caribbean woman	52-year-old Caucasian man	55-year-old Caucasian woman	30-year-old Caucasian woman	30-year-old Caucasian woman
Comorbidities	None	None	None	None	None	COPD	Cardiovascular comorbidities	None
Characteristics of the initial attack	Absence of clinically concerning symptoms, without motor impairment and/or ataxia	Absence of clinically concerning symptoms, without motor impairment and/or ataxia	Presence of clinically concerning symptoms, with motor impairment and/or ataxia	Absence of clinically concerning symptoms, without motor impairment and/or ataxia	Absence of clinically concerning symptoms, without motor impairment and/or ataxia	Presence of clinically concerning symptoms, with motor impairment and/or ataxia	Absence of clinically concerning symptoms, without motor impairment and/or ataxia	Presence of clinically concerning symptoms, with motor impairment and/or ataxia
Cognitive impairment	Absent	Absent	Absent	Present	Present	Absent	Present	Absent
Brain lesion load	Low	Low	Moderate	Low	High	Moderate	Moderate	High
Number of infratentorial lesions on brain MRI	0	0	1	0	3	0	1	2
Number of lesions on spinal cord MRI	0	1	0	0	1	2	0	6
Number of Gd-enhancing lesions on brain MRI	1	1	1	3	1	2	4	1

*COPD* chronic obstructive pulmonary disease*, Gd* gadolinium, *MRI* magnetic resonance imaging

A series of additional scenarios were presented where, for each clinical case, one single parameter (i.e. a patient characteristic) changed at a time (e.g. all parameters held constant but now instead of having no infratentorial lesions, the patient has 1 infratentorial lesion). The parameters susceptible to change were those that, if different, could impact the treatment approach due to an increased or decreased risk of disease/disability progression. These included the ethnicity of the patient (Caucasian, yes vs no) [28–31], comorbidities (yes vs no) [32], cognitive impairment (yes vs no) [33], a previous attack in the past 12 months (i.e. at least two attacks within the first 12 months of the disease; yes vs no) [2, 34], motor impairment and/or ataxia (yes vs no) [35], brain lesion load (low, moderate, high) [36, 37], number of infratentorial lesions (none, 1, 2, ≥3) [38–40], number of spinal cord lesions (none, 1, 2, ≥3) [36, 39, 41], and number of gadolinium‑enhancing lesions (0-2, ≥3) [41] (Table 2). These categorisations were defined based on the steering committee’s assessment of their potential influence on the decision to recommend monoclonal HET. Aspects related to tolerance and safety of the relevant treatments were not explicitly addressed in the proposed clinical scenarios. There was a total of 108 statements. In the same way as for the original scenarios, the participants were asked to state if, for each of these iterations, they would recommend monoclonal HET.

**Table 2:** Single-parameter variations tested in the Delphi questionnaire used to explore expert consensus and identify key factors driving the initiation of monoclonal high-efficacy therapies amongst treatment-naive relapsing-remitting multiple sclerosis patients, France

Parameters tested
**Patient is Caucasian** (yes vs no)
**Presence of comorbidities** (yes vs no)
**Cognitive impairment**
Presence of cognitive impairment
Absence of cognitive impairment
**Presence of a previous attack in the past 12 months** (yes vs no)
**Motor impairment and/or ataxia**
Presence of motor impairment and/or ataxia
Absence of motor impairment and/or ataxia
**Brain lesion load**
Low (<10 lesions)
Moderate (10-20 lesions)
High (>20 lesions)
**Number of infratentorial lesions on brain MRI**
0 lesions
1 lesion
2 lesions
≥3 lesions
**Number of lesions on spinal cord MRI**
0 lesions
1 lesion
2 lesions
≥3 lesions
**Number of Gd-enhancing lesions on brain MRI**
0-1-2 lesion(s)
≥3 lesions

*Gd* gadolinium, *MRI* magnetic resonance imaging

All instructions, questionnaire statements and possible answers, and the discussion support materials were written in French. The information presented in the tables, figures and online resources are translations of the original content, using standard and consistent medical English terminology.

### Delphi rounds

This study design considered two rounds of questionnaires (Fig. 1). A first round of questionnaires was sent online via personalised e-mail invitations. To fill out the questionnaire, each expert from the panel had to use an individual code they had received. Data from participants responses was anonymised and only accessible to the steering committee in an aggregated format.

After the data from the first round were analysed, a national e-workshop with the participants was organised to: i) present the results of the first round to the participants, ii) discuss the topics where consensus was not reached, in the light of the available literature at the time. The members of the steering committee presented the clinical cases, the statements where consensus was not reached and led the discussion with the expert panel. Out of the 31 participating experts who responded to the questionnaire, 27 attended the e-workshop. The discussion minutes of the workshop were sent via email to the 4 members of the expert panel who could not be present.

After the meeting, a second round of questionnaires, containing the questions that did not achieve consensus the first time, were sent via email to each participating expert (same procedures as for the first round). Participants were free to maintain or change the responses they had given for each scenario during the first round.

### Consensus criteria

Consensus for agreement with each of the statements (i.e. consensus in favour of the statements) was defined by a median score of ≥4 and ≥80% of ratings in the 4 to 5 range. The agreement could be either strong or moderate: ‘Strong agreement’ was defined as ≥90% of the responses being rated at 4 or 5; and ‘Moderate agreement’ was defined as ≥80% (and <90%) of the responses being rated at 4 or 5. Likewise, consensus for the disagreement with a statement was reached if the median score was ≤2 and there were ≥80% of ratings in the 1 to 2 range. ‘Strong disagreement’ with the statements was defined as ≥90% of the responses rated 1 or 2; and ‘Moderate disagreement’ was defined as ≥80% (and <90%) of the responses being rated 1 or 2.

### Data analysis and factors influencing experts’ decisions

For each statement, the distribution of ratings is presented using percentages. Based on the responses, the factors influencing experts’ decision to initiate monoclonal HET were identified. Lastly, based on the study results, a framework grounded in these factors is proposed.

## Results

For both rounds of the study, 31 responses were received out of the 31 questionnaires sent out to experts.

Following completion of both rounds, of the 108 statements evaluated, 70 (64.8%) achieved strong consensus in favour of monoclonal HET initiation (≥90%), 18 (16.7%) reached moderate consensus (≥80 and <90%), 19 (17.6%) did not reach consensus and 1 (0.9%) reached strong consensus not to initiate monoclonal HET (≥90%).

### Round 1 results

From the 108 statements sent for rating in the first round, 74 statements (68.5%) reached consensus. Amongst these 74 statements, 58 reached ‘strong agreement’ in favour of initiating monoclonal HET (i.e. ≥90% of responses were rated at 4 or 5). Overall, cases with a more severe presentation either clinically or radiologically were more likely to render consensus for the start of a monoclonal HET amongst the expert panel (e.g. clinical cases 5 to 8, original). In line with this, a non-concerning clinical presentation and imaging findings prompted the experts to reach consensus towards no monoclonal HET initiation (clinical case 1: disagreement with monoclonal HET; i.e. ≥80% of responses rated at 1 or 2). Online Resource 2 shows the consensus reached in favour or against monoclonal HET initiation according to a proposed severity classification of the clinical cases. The results from Round 1 are detailed in Online Resource 3.

### Round 2 results

In Round 2, consensus was reached for 15 out of the 34 statements (44.1%) for which consensus had not been reached in Round 1. Of these, 12 reached ‘strong agreement’. In total, after the two rounds of questionnaires, consensus was reached for 89/108 (82.4%) statements. A ‘strong agreement’ was reached for 71 of these statements. The final results can be found in Table 3.

**Table 3:** Final results following the Round 2 of the Delphi study conducted in France used to explore expert consensus and identify key factors driving the initiation of monoclonal HET amongst treatment-naïve relapsing-remitting multiple sclerosis patients

N.	Question: Would you recommend initiating a monoclonal HET for this patient, if…?	Most frequent answer; consensus level (%)			Median
		Lack of consensus (<80%)	Moderate consensus (80-90%)	Strong consensus (≥90%)	
*Clinical case 1: 25-year-old Caucasian woman without comorbidities, presenting with an initial attack without motor impairment or ataxia, and without cognitive impairment. Brain MRI shows a low brain lesion load, with no infratentorial lesions, no spinal cord involvement, and 1 Gd-enhancing lesion.*					
Q1	Presentation upon initial profile		No (86%)		1
Q2	Presence of cognitive impairment	Yes (45%)			3
Q3	Presence of a previous attack in the past 12 months	Yes (77%)			4
Q4	Presence of motor impairment and/or ataxia	Yes (71%)			4
Q5	Presence of a moderate brain lesion load on brain MRI	Yes (58%)			4
Q6	Presence of a high brain lesion load on brain MRI		Yes (80%)		5
Q7	Presence of 1 infratentorial lesion	Yes (39%)			3
Q8	Presence of 2 infratentorial lesions	Yes (77%)			4
Q9	Presence of ≥3 infratentorial lesions			Yes (94%)	5
Q10	Presence of 1 spinal cord lesion	Yes (61%)			4
Q11	Presence of 2 spinal cord lesions			Yes (90%)	5
Q12	Presence of ≥3 spinal cord lesions			Yes (97%)	5
Q13	Presence of ≥3 Gd-enhancing lesions		Yes (81%)		5
*Clinical case 2: 32-year-old Maghrebi man without comorbidities, presenting with an initial attack without motor impairment or ataxia, and without cognitive impairment. Brain MRI shows a low brain lesion load, with no infratentorial lesions, 1 spinal cord lesion, and 1 Gd-enhancing lesion.*					
Q1	Presentation upon initial profile	Yes (65%)			4
Q2	The patient is not of Maghrebi origin	No (42%)			3
Q3	Presence of cognitive impairment			Yes (94%)	5
Q4	Presence of a previous attack in the past 12 months		Yes (86%)		5
Q5	Presence of motor impairment and/or ataxia			Yes (90%)	4
Q6	Presence of a moderate brain lesion load on brain MRI		Yes (83%)		4
Q7	Presence of a high brain lesion load on brain MRI			Yes (96%)	5
Q8	Presence of 1 infratentorial lesion		Yes (80%)		4
Q9	Presence of 2 infratentorial lesions		Yes (86%)		5
Q10	Presence of ≥3 infratentorial lesions			Yes (90%)	5
Q11	Absence of spinal cord lesions	No (74%)			2
Q12	Presence of 2 spinal cord lesions			Yes (90%)	5
Q13	Presence of ≥3 spinal cord lesions			Yes (97%)	5
Q14	Presence of ≥3 Gd-enhancing lesions		Yes (86%)		5
*Clinical case 3: 27-year-old Caucasian man without comorbidities, presenting with an initial attack with motor impairment and/or ataxia, and without cognitive impairment. Brain MRI shows a moderate brain lesion load, with 1 infratentorial lesion, no spinal cord involvement, and 1 Gd-enhancing lesion.*					
Q1	Presentation upon initial profile	Yes (77%)			4
Q2	Presence of cognitive impairment			Yes (97%)	5
Q3	Presence of a previous attack in the past 12 months		Yes (87%)		5
Q4	Absence of motor impairment and/or ataxia	Yes (39%)			3
Q5	Presence of a low brain lesion load on brain MRI	Yes (52%)			4
Q6	Presence of a high brain lesion load on brain MRI			Yes (90%)	5
Q7	Absence of infratentorial lesions	Yes (45%)			3
Q8	Presence of 2 infratentorial lesions			Yes (90%)	5
Q9	Presence of ≥3 infratentorial lesions		Yes (87%)		5
Q10	Presence of 1 spinal cord lesion		Yes (86%)		4
Q11	Presence of 2 spinal cord lesions			Yes (93%)	5
Q12	Presence of ≥3 spinal cord lesions			Yes (100%)	5
Q13	Presence of ≥3 Gd-enhancing lesions			Yes (94%)	5
*Clinical case 4: 35-year-old Afro-Caribbean woman without comorbidities, presenting with an initial attack without motor impairment or ataxia, with cognitive impairment. Brain MRI shows a low brain lesion load, with no infratentorial lesions, no spinal cord involvement, and 3 Gd-enhancing lesions.*					
Q1	Presentation upon initial profile		Yes (87%)		4
Q2	The patient is not of Afro-Caribbean origin	Yes (68%)			4
Q3	Absence of cognitive impairment	Yes (77%)			4
Q4	Presence of a previous attack in the past 12 months			Yes (90%)	5
Q5	Presence of motor impairment and/or ataxia		Yes (87%)		5
Q6	Presence of a moderate brain lesion load on brain MRI			Yes (90%)	4
Q7	Presence of a high brain lesion load on brain MRI			Yes (97%)	5
Q8	Presence of 1 infratentorial lesion			Yes (100%)	5
Q9	Presence of 2 infratentorial lesions			Yes (90%)	5
Q10	Presence of ≥3 infratentorial lesion			Yes (100%)	5
Q11	Presence of 1 spinal cord lesion			Yes (94%)	4
Q12	Presence of 2 spinal cord lesions			Yes (96%)	5
Q13	Presence of ≥3 spinal cord lesions			Yes (100%)	5
Q14	Presence of 0-1-2 Gd-enhancing lesion(s)	Yes (52%)			4
*Clinical case 5: 52-year-old Caucasian man without comorbidities, presenting with an initial attack without clinically concerning symptoms, and without motor impairment or ataxia, with cognitive impairment. Brain MRI shows a high brain lesion load, with 3 infratentorial lesions, 1 spinal cord lesion, and 1 Gd-enhancing lesion.*					
Q1	Presentation upon initial profile		Yes (83%)		5
Q2	Absence of cognitive impairment		Yes (83%)		5
Q3	Presence of a previous attack in the past 12 months			Yes (97%)	5
Q4	Presence of motor impairment and/or ataxia			Yes (93%)	5
Q5	Presence of a low brain lesion load on brain MRI			Yes (100%)	5
Q6	Presence of a moderate brain lesion load on brain MRI			Yes (100%)	5
Q7	Absence of infratentorial lesions			Yes (100%)	5
Q8	Presence of 1 infratentorial lesion			Yes (100%)	5
Q9	Presence of 2 infratentorial lesions		Yes (83%)		5
Q10	Absence of spinal cord lesions			Yes (100%)	5
Q11	Presence of 2 spinal cord lesions			Yes (93%)	5
Q12	Presence of ≥3 spinal cord lesions			Yes (100%)	5
Q13	Presence of ≥3 Gd-enhancing lesions			Yes (100%)	5
*Clinical case 6: 55-year-old Caucasian woman with COPD, presenting an initial attack with motor impairment and/or ataxia, without cognitive impairment. Brain MRI shows a moderate brain lesion load, with no infratentorial lesions, 2 spinal cord lesions, and 2 Gd-enhancing lesions.*					
Q1	Presentation upon initial profile			Yes (93%)	5
Q2	Absence of comorbidities			Yes (96%)	5
Q3	Presence of cognitive impairment			Yes (97%)	5
Q4	Presence of a previous attack in the past 12 months			Yes (100%)	5
Q5	Absence of motor impairment and/or ataxia			Yes (100%)	5
Q6	Presence of a low brain lesion load on brain MRI		Yes (83%)		5
Q7	Presence of a high brain lesion load on brain MRI			Yes (96%)	5
Q8	Presence of 1 infratentorial lesion			Yes (97%)	5
Q9	Presence of 2 infratentorial lesions			Yes (97%)	5
Q10	Presence of ≥3 infratentorial lesions			Yes (100%)	5
Q11	Absence of spinal cord lesions	Yes (74%)			3
Q12	Presence of 1 spinal cord lesion			Yes (97%)	5
Q13	Presence of ≥3 spinal cord lesions			Yes (100%)	5
Q14	Presence of ≥3 Gd-enhancing lesions			Yes (100%)	5
*Clinical case 7: 30-year-old Caucasian woman with cardiovascular comorbidities, presenting with an initial attack without motor impairment or ataxia, with cognitive impairment. Brain MRI shows a moderate brain lesion load with 1 infratentorial lesion, no spinal cord involvement, and 4 Gd-enhancing lesions.*					
Q1	Presentation upon initial profile			Yes (97%)	5
Q2	Absence of comorbidities			Yes (97%)	5
Q3	Absence of cognitive impairment			Yes (90%)	5
Q4	Presence of a previous attack in the past 12 months			Yes (100%)	5
Q5	Presence of motor impairment and/or ataxia			Yes (100%)	5
Q6	Presence of a low brain lesion load on brain MRI		Yes (87%)		5
Q7	Presence of a high brain lesion load on brain MRI			Yes (100%)	5
Q8	Absence of infratentorial lesions		Yes (86%)		4
Q9	Presence of 2 infratentorial lesions			Yes (100%)	5
Q10	Presence of ≥3 infratentorial lesions			Yes (100%)	5
Q11	Presence of 1 spinal cord lesion			Yes (100%)	5
Q12	Presence of 2 spinal cord lesion			Yes (100%)	5
Q13	Presence of ≥3 spinal cord lesions			Yes (100%)	5
Q14	Presence of 0-1-2 Gd-enhancing lesion(s)	Yes (58%)			4
*Clinical case 8: 30-year-old Caucasian woman without comorbidities, presenting with an initial attack with motor impairment and/or ataxia, without cognitive impairment. Brain MRI shows a high brain lesion load, with 2 infratentorial lesions, 6 spinal cord lesions, and 1 Gd-enhancing lesion.*					
Q1	Presentation upon initial profile			Yes (97%)	5
Q2	Presence of cognitive impairment			Yes (100%)	5
Q3	Presence of a previous attack in the past 12 months			Yes (100%)	5
Q4	Absence of motor impairment and/or ataxia			Yes (96%)	5
Q5	Presence of a low brain lesion load on brain MRI			Yes (97%)	5
Q6	Presence of a moderate brain lesion load on brain MRI			Yes (96%)	5
Q7	Absence of infratentorial lesions			Yes (97%)	5
Q8	Presence of 1 infratentorial lesion			Yes (96%)	5
Q9	Presence of ≥3 infratentorial lesions			Yes (100%)	5
Q10	Absence of spinal cord lesions			Yes (93%)	5
Q11	Presence of 1 spinal cord lesion			Yes (94%)	5
Q12	Presence of 2 spinal cord lesions			Yes (97%)	5
Q13	Presence of ≥3 Gd-enhancing lesions			Yes (100%)	5

*COPD* chronic obstructive pulmonary disease*, Gd* gadolinium*, HET* high-efficacy therapy*, MRI* magnetic resonance imaging

### Factors leading to consensus in favour of monoclonal HET initiation

The analysis identified factors that led the expert panel to consensus towards recommending monoclonal HET initiation. Factors with a ‘strong influence on experts’ decision to initiate monoclonal HET’ were parameters whose presence systematically led to consensus in favour of monoclonal HET initiation. Factors with an ‘intermediate influence on experts’ decision to initiate monoclonal HET’ were parameters whose presence led to consensus towards monoclonal HET initiation in most of the cases (except for ‘Clinical case 1’).

Factors classified as having a ‘strong influence on experts’ decision to initiate monoclonal HET’ were radiological markers of high disease activity and included a high brain lesion load (>20 lesions), ≥2 spinal cord lesions, ≥3 gadolinium-enhancing lesions and ≥3 infratentorial lesions. Factors considered to have an ‘intermediate influence on experts’ decision to initiate monoclonal HET’ included a moderate brain lesion load (10–20 lesions), an initial attack with clinically concerning symptoms, a single spinal cord lesion, 1 or 2 infratentorial lesions, a previous attack in the past 12 months and cognitive impairment.

### Framework of factors influencing treatment decisions

**Fig 2:**
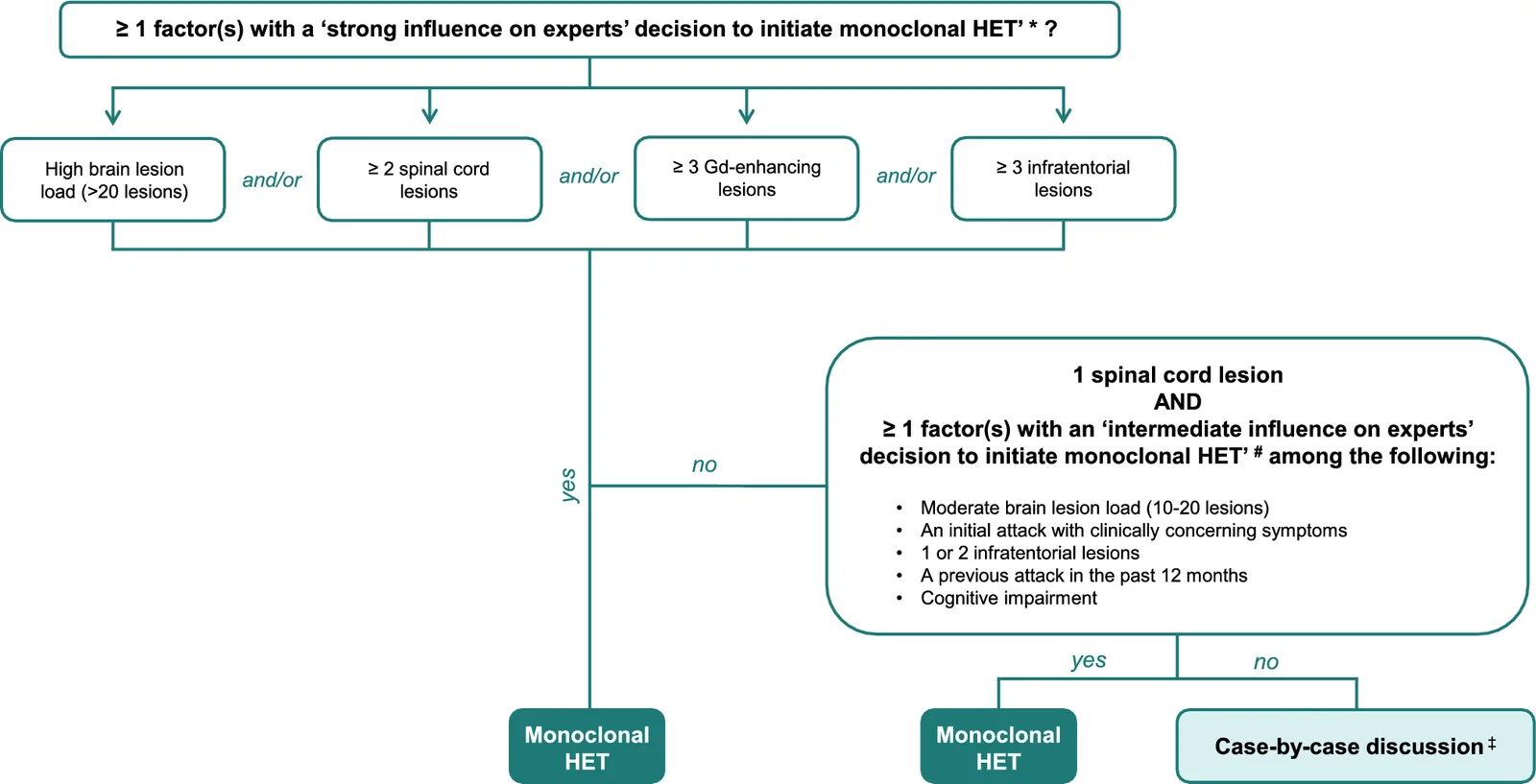
Proposed framework based on factors leading to experts’ decision to recommend initiating monoclonal HET amongst treatment-naïve relapsing-remitting multiple sclerosis patients. * Factors with a ‘strong influence on experts’ decision to initiate monoclonal HET’ were parameters whose presence systematically led to consensus in favour of monoclonal HET initiation. ^#^ Factors with an ‘intermediate influence on experts’ decision to initiate monoclonal HET’ were parameters whose presence led to consensus towards monoclonal HET initiation in most of the cases (except for ‘Clinical case 1’). ^‡^ Other factors with apparently less influence on monoclonal HET initiation (older age, male sex, comorbidities, and non-Caucasian ethnicity), insufficiently evaluated in this Delphi process, may also be considered alongside imagining and non-imaging variables during treatment discussion

Based on the analysis of the factors leading to consensus to recommend monoclonal HET in the eight clinical cases, the authors would like to propose a framework (Fig. 2) based on the analysis of the data gathered through this Delphi study. In the presence of one or more factors with a ‘strong influence on experts’ decision to initiate monoclonal HET’, the initiation of monoclonal HET would be advisable. If there are no such factors present for a given patient, the presence of a single spinal cord lesion would merit monoclonal HET initiation if there were one or more other factors with an ‘intermediate influence on experts’ decision to initiate monoclonal HET’. If no factors of strong or intermediate influence on experts’ decision to initiate monoclonal HET are present, then it would be advisable to discuss the therapeutic approach under the light of demographic and clinical history factors, such as sex, age, ethnicity and comorbidities.

## Discussion

### Summary of the results

The present study aimed to gauge consensus via the Delphi method regarding the use of monoclonal HET (anti-CD20 and anti-α4-integrin monoclonal antibodies) for treatment-naïve RRMS patients amongst an expert panel of neurologists from France. The study found that, overall, clinical case severity led to consensus to start monoclonal HET. Notably, the factors with a ‘strong influence on experts’ decision to initiate monoclonal HET’, that systematically led to consensus towards recommending monoclonal HET, were a high brain lesion load, ≥2 spinal cord lesions, ≥3 gadolinium-enhancing lesions and ≥3 infratentorial lesions. The factors that often—but not always—led to consensus towards recommending monoclonal HET initiation (labelled as factors with an ‘intermediate influence on experts’ decision to initiate monoclonal HET’) were a moderate brain lesion load, an initial attack with clinically concerning symptoms, a single spinal cord lesion, 1 or 2 infratentorial lesions, a previous attack in the past 12 months and cognitive impairment.

Before reflecting on the results of this study, it is important to acknowledge that both findings and the proposed treatment algorithm should be interpreted in the context of the eight clinical cases used for this Delphi process. Additionally, the cut-offs for the different number of lesions leading to a recommendation for monoclonal HET initiation reflected the exploratory nature of the Delphi process and may not align with other cut-offs described in the literature. Different studies have applied different cut-offs, and some suggest that even singular lesions have been associated with poorer prognosis [36, 39, 41, 42]. In the absence of consensus on these thresholds, the steering committee selected the thresholds presented in Table 3 which were considered relevant for real-world clinical practice, to be further evaluated in the Delphi process. Lastly, it should be noted that the clinical cases presented are hypothetical scenarios with multiple iterations, and not a representative sample of patients seen in everyday practice. Consequently, the ‘frequency of monoclonal HET initiation’ or the ‘proportion of cases in which monoclonal HET is recommended’ cannot be estimated from these findings.

### Factors leading to consensus to advise monoclonal HET

Overall, the factors that were associated with a systematic expert recommendation of monoclonal HET are characteristics indicative of higher disease severity. This observation is in line with what would be expected, as a more aggressive presentation at disease onset is associated with poor prognosis, particularly an increased risk of irreversible disability [43]. Before discussing the factors leading to consensus in detail, it is important to note that the increased use of HET, both in clinical practice and these hypothetical scenarios, is linked to an increase in the evidence supporting a favourable benefit–risk profile [3, 4], as well as to the ability to monitor and mitigate certain risks. For example, the introduction of the anti-JC virus antibody index (JCV index) as a risk-stratification tool for progressive multifocal leukoencephalopathy [44] appears to have translated into a reduction of the incidence of this event in real-world patients treated with anti-α4-integrin monoclonal antibody [45]. If the existing evidence and monitoring tools were not available, it is likely that experts that took part in this Delphi consensus would not have reached similar conclusions. More broadly, safety and feasibility considerations regarding monoclonal HET initiation decision-making encompass, in addition to the JCV index (PML risk), other factors such as infection history, immunoglobulin levels, vaccination status, pregnancy plans, patient preference, amongst others. Whilst these factors were outside the scope of the present study, they remain important to consider in clinical practice.

An insight that warrants particular attention is the fact that all the factors with a ‘strong influence on experts’ decision to initiate monoclonal HET’ were variables derived from imaging results. This illustrates the major role of imaging when conducting patient risk assessment and deciding on the therapeutic approach [36–41, 43]. However, it is also interesting to note that clinical factors—which are essential for a holistic patient evaluation—did not systematically lead to a monoclonal HET recommendation (thus, they were classified as having only an ‘intermediate’ influence on experts’ decision to initiate monoclonal HET). This result may either be a function of other patient characteristics in a relatively limited number of potential scenarios, or a manifestation of a modern medicine paradigm that relies more on technology for determining the therapeutic approach. We recommend maintaining a thoughtful stance towards these findings and allowing them to inform further discussion.

There is an important body of evidence showcasing how imaging findings are associated with the progression of disease [36–41, 43]. For example, Brownlee and colleagues analysed a cohort of 178 patients initially diagnosed with a clinically isolated syndrome to determine early MRI predictors of long-term outcomes, such as the development of secondary progressive MS and disability [41]. The authors found that spinal cord lesions and evidence of active inflammation (gadolinium-enhancing lesions) were consistently associated with poorer outcomes. Comparably, results from a Dutch longitudinal study showed that spinal cord lesions at the baseline patient evaluation were highly predictive of disability for up to 11 years of follow-up [39]. This cohort study also found infratentorial lesions to predict disability in the long-term. Lastly, regarding brain lesion load, a prospective cohort study from Barcelona found that a higher number of T2 lesions was associated with earlier irreversible disability [37].

Factors having an ‘intermediate influence on experts’ decision to initiate monoclonal HET’ included not only imaging markers signalling damage, but also clinical characteristics of the patients. It is important to note that all these factors do not have the same clinical significance nor the same effect on the patient prognosis. Therefore, their grouping in a single category responds to the categorisation followed for this Delphi exercise and should be appraised with caution. For example, the presence of cognitive impairment or a previous attack in the past 12 months (therefore, having had at least two attacks within the first 12 months of the disease) are standalone markers of concern [34, 46, 47]. Another important aspect of this Delphi process is the evolution of expert responses between rounds following the structured discussion. The e-workshop provided an opportunity to collectively reassess the relevance of certain factors, particularly cognitive impairment and infratentorial lesions, which appeared to be initially underestimated. This likely contributed to the increase in consensus observed in the second round and highlights the dynamic nature of expert-based decision-making.

Regarding cognitive impairment as a factor with an ‘intermediate influence on experts’ decision to initiate monoclonal HET’, an Austrian study following almost 800 RRMS patients found that patients presenting cognitive dysfunction at disease onset had approximately a fourfold risk of severe disability in comparison with patients with a preserved cognitive function [46]. Similarly, Cavaco and colleagues found that patients with cognitive dysfunction were more likely to develop a progressive disease course and had lower survival over a follow-up period of up to 16 years [47]. Regarding having had a previous attack in the past 12 months, upon reviewing the results, the members of the steering committee agreed that two attacks in a window of 12 months would likely always prompt them to initiate HET. This is not reflected as such in the results (nor in the therapeutic decision framework) presumably due to particularities of the clinical cases presented. Evidence from a prospective cohort in Australia suggests that early relapses (in the first two and a half years since disease onset) were strong predictors of disability worsening [34]. Lastly, with respect to motor impairment, Malpas and colleagues followed up a large cohort of MS patients and found that concerning symptoms such as pyramidal signs or early disability after the first attack had considerable predictive value for future disability [35].

Demographic and clinical history characteristics did not seem to lead the experts to advise HET. These findings should be interpreted with caution, as there were very few cases where the patients were not Caucasian or had comorbidities. Whilst there is evidence suggesting that, for example, Afro-Caribbean [28] and North-African [29–31] patients may have a poorer prognosis than patients of White European descent, these and other demographic factors seemed not to justify monoclonal HET initiation on their own in this Delphi exercise. As proposed in Fig. 2, these factors should be considered alongside clinical and imaging variables to decide the best individualised treatment approach.

Lastly, although not evaluated in this Delphi study, there are emerging biomarkers and imaging features that may be relevant predictors of the MS clinical course, inflammatory activity and risk of disability accumulation, such as the levels of serum neurofilament light chains (NfL) [48–50], serum glial fibrillary acidic protein (GFAP) [51–53], cerebrospinal fluid (CSF) oligoclonal bands [54], CSF kappa free light chains [55], paramagnetic rim lesions (PRL) [56, 57], cortical lesions [58], brain and spinal cord atrophy [59], optic nerve involvement and optical coherence tomography-derived measures [60]. Furthermore, some evidence suggests that, for example, monoclonal HET initiation can lower disability accrual amongst patients with high levels of NfL [50]. Whilst these biomarkers show promise, most are not yet part of the standard clinical practice. It is therefore important to reflect on how should they enter routine clinical practice, they may influence future therapeutic strategies and inform clinical decision-making, including the initiation of a monoclonal HET rather than a moderate-efficacy therapy.

### Limitations and strengths

It is important to discuss certain caveats associated with the methods chosen for this study. First, there are certain biases inherent to the Delphi methodology used [25], that potentially arose when changes to patients’ characteristics were proposed. Notably, cognitive biases may give higher weights to the importance of the single ‘changing factor’ (e.g. ethnic group, changing from ‘Afro-Caribbean’ to ‘Caucasian’). This change alone could make the Delphi participant to focus momentarily but disproportionately on the characteristic that is being changed for that specific scenario. Additionally, real-world treatment decisions tend to be based on a comprehensive assessment of clinical and imaging activity, sociodemographic factors, comorbidities, and treatment characteristics, as well as the potential interactions between these factors. Therefore, varying one parameter at a time may fail to capture the combined or interactive effect of these factors, and is likely to influence the recommended therapeutic course of action in a way that may be different from what might occur in routine clinical practice, where a single factor would not be appraised in isolation.

Second, the degree of clinical recovery after the first neurological episode (initial attack) of the disease was not presented as a characteristic to be considered amongst the clinical cases used for this exercise. Whilst an incomplete recovery has been associated with poorer prognosis [61], the steering committee decided not to include it for two reasons: (i) to limit the degree of heterogeneity between patients, maintaining a relative parsimony in the characteristics presented, and (ii) to focus on the information that is likely to be available at treatment initiation in a patient that is yet to receive any DMT (a patient will likely start a DMT before the ‘recovery’ status for the first attack is fully known).

Third, as previously mentioned, there are some emerging serum, CSF, and imaging biomarkers [48–60] that have not been included in the clinical cases presented to the experts but may be relevant in the modern or future treatment decision-making. Prior to the Delphi exercise, the steering committee assessed all potential factors influencing treatment decisions and decided not to include these markers to: i) maintain a relative parsimony in the data presented for each clinical case, and to ii) focus on factors that are available in routine clinical practice across most clinical settings. The authors believe that it will be important to include these factors in similar future studies, once they are widely incorporated as part of the routine patient assessment for treatment decisions.

Fourth, having a second round of ratings after a workshop where the clinical cases and the current literature were discussed may have changed the perspective of some members of the expert panel. This may have produced differing results between rounds. For example, in the case of clinical case 3, having 2 infratentorial lesions prompted ‘strong agreement’ to initiate monoclonal HET, but having ≥3 infratentorial lesions prompted only ‘moderate agreement’. This apparently counter-intuitive finding is because consensus was reached for ≥3 lesions—but not for 2 lesions—during Round 1. Therefore, when the case with 2 lesions was presented to the expert panel again in Round 2, after having discussed the literature and the impact of timely monoclonal HET initiation for such cases, ‘strong consensus’ was reached. This may not be a limitation *per se*, as it is an inherent characteristic of the iterative Delphi methodology. However, it is important to consider its potential implications when interpreting the results.

Fifth, it is Important to explicitly state that the proposed framework depicts the factors that influenced the decision to recommend monoclonal HET amongst members of this expert panel and it should not be used as a clinical guideline. In order for these results to further inform any recommendations or guidelines, they should be ideally validated with empirical evidence.

There are several strengths that should be noted as well. First, this consensus exercise brought together a diverse sample of neurologists from different healthcare settings and regions across France, thus capturing a broad base of clinical expertise. Second, the study followed a structured Delphi process, which strengthens the reliability in the consensus reached. Third, anonymous responses have likely allowed participants to express their honest perspectives, limiting desirability and other types of response biases.

## Conclusion

The consensus amongst an expert panel of French neurologists was to systematically recommend monoclonal HET initiation amongst treatment-naïve RRMS patients that had high disease activity on imaging, including a high brain lesion load, ≥2 spinal cord lesions, ≥3 gadolinium-enhancing lesions and ≥3 infratentorial lesions. The factors that often—but not always—led to expert consensus to advise monoclonal HET were a moderate brain lesion load, an initial attack with clinically concerning symptoms, a single spinal cord lesion, 1 or 2 infratentorial lesions, a previous attack in the past 12 months and cognitive impairment. This Delphi study suggests that monoclonal HET initiation is primarily driven by imaging markers of high disease activity, which showed the strongest influence on treatment decisions. In contrast, clinical features and some MRI findings had a more variable and moderate impact. Amongst these, the presence of a single spinal cord lesion appeared to carry the greatest weight. These findings may help inform individualised therapeutic strategies, pending results from ongoing randomised trials.

## Supplementary Information

Below is the link to the electronic supplementary material.

Supplementary file1 (DOCX 4813 KB)

Supplementary file2 (DOCX 18 KB)

Supplementary file3 (DOCX 42 KB)

## Data Availability

The corresponding author, who had complete access to all aggregated voting data collected during the two Delphi rounds, assumes responsibility for the integrity of the data and the accuracy of the analysis. An anonymized dataset including the individual voting results of the panel members for both rounds can be made available from the corresponding author upon reasonable request.
