# Supplementary material for: DELPHI expert consensus-derived factors influencing the early use of monoclonal high-efficacy therapies in treatment-naïve patients with relapsing-remitting multiple sclerosis
Source: J Neurol. 2026 Oct 22;273(11):679. doi: 10.1007/s00415-026-14167-4 (PMC13690885; doi:10.1007/s00415-026-14167-4)
Supplement: Supplementary file 1 [file 415_2026_14167_MOESM1_ESM.docx]

**DELPHI expert consensus-derived factors influencing the early use of monoclonal high-efficacy therapies in treatment-naïve patients with relapsing-remitting multiple sclerosis**

Journal : Journal of Neurology

Bertrand Bourre^1^, Olivier Guerrier^2^, Xavier Ayrignac^3^, Laurent Suchet^4^, Jérôme de Seze^5^, Ines Masmoudi^6^, Vito AG Ricigliano^7^, Patrick Vermersch^8^, Jonathan Ciron^9^ on behalf of the CAReSEP working group.

^1^ CHU Rouen, Department of Neurology and Inserm CIC-CRB 1404, F-76000, Rouen, France

^2^ Department of Neurology and Neurovascular Unit, Valence Hospital, Valence, France

^3^ Univ Montpellier, INM, INSERM, Département de Neurologie, CRC sclérose en plaques, CRMR LEUKOFRANCE & Centre d’investigation clinique U1411, CHU de Montpellier, Montpellier, France

^4^ Department of Neurology, European Hospital of Marseille, Marseille, France

^5^ Department of Neurology and Clinical Investigation Center, Strasbourg University Hospital, Strasbourg, France

^6^ Department of Neurology, Amiens University Hospital, Amiens, France

^7^ Neurology Unit, GHNE Paris Saclay Hospital, Orsay, France; Université Paris-Saclay, UNIACT, Neurospin – INSERM UMR 1129, CEA, Gif-sur-Yvette, France

^8^ University of Lille, INSERM U1172 LilNCog, Lille University Hospital, FHU Precise, Lille, France

^9^ Department of Neurology, CRC-SEP, Toulouse University Hospital, Toulouse, France; INSERM UMR1291 – CNRS UMR5051, INFINITY, University Toulouse III, Toulouse, France

**Corresponding author:**

Jonathan Ciron

[ciron.j@chu-toulouse.fr](mailto:ciron.j@chu-toulouse.fr)

**Online Resource 1** MRI scans used to classify low, moderate and high brain lesion load in the Delphi study to explore expert consensus & identify key factors driving the initiation of monoclonal high-efficacy therapies among treatment-naive relapsing-remitting multiple sclerosis patients, France

| **Low brain lesion load (<10 lesions)** | | | | | | | | **Moderate brain lesion load (10-20 lesions)** | | | | | | | | | | **High brain lesion load (>20 lesions)** | | | | | | | | |
| --- | --- | --- | --- | --- | --- | --- | --- | --- | --- | --- | --- | --- | --- | --- | --- | --- | --- | --- | --- | --- | --- | --- | --- | --- | --- | --- |
| *Example 1* | | | | | | | | *Example 1* | | | | | | | | | | *Example 1* | | | | | | | | |
|  | |  | |  | |  | |  | |  | | |  | | |  | |  |  | | |  | | |  | |
| *Exemple 2* | | | | | | | | *Exemple 2* | |  | | |  | | |  | | *Exemple 2* |  | | |  | | |  | |
|  | | | |  | | | |  | | |  | | | |  | | |  |  | | |  | | |  | |
| *Exemple 3* | | | | | | | | *Exemple 3* | | | | | | | | | | *Exemple 3* | | | | | | | | |
|  | | | | | | | |  | |  | | |  | | |  | |  | | |  | | |  | | |
| *Exemple 4* | | | | | | | | *Exemple 4* | |  | | |  | | |  | | *Exemple 4* | | |  | | |  | | |
|  |  | |  | |  | |  |  |  | | |  | |  | | |  |  | |  | | |  | | |  |
