# Supplementary material for: DELPHI expert consensus-derived factors influencing the early use of monoclonal high-efficacy therapies in treatment-naïve patients with relapsing-remitting multiple sclerosis
Source: J Neurol. 2026 Oct 22;273(11):679. doi: 10.1007/s00415-026-14167-4 (PMC13690885; doi:10.1007/s00415-026-14167-4)
Supplement: Supplementary file 2 [file 415_2026_14167_MOESM2_ESM.docx]

**DELPHI expert consensus-derived factors influencing the early use of monoclonal high-efficacy therapies in treatment-naïve patients with relapsing-remitting multiple sclerosis**

Journal : Journal of Neurology

Bertrand Bourre^1^, Olivier Guerrier^2^, Xavier Ayrignac^3^, Laurent Suchet^4^, Jérôme de Seze^5^, Ines Masmoudi^6^, Vito AG Ricigliano^7^, Patrick Vermersch^8^, Jonathan Ciron^9^ on behalf of the CAReSEP working group.

^1^ CHU Rouen, Department of Neurology and Inserm CIC-CRB 1404, F-76000, Rouen, France

^2^ Department of Neurology and Neurovascular Unit, Valence Hospital, Valence, France

^3^ Univ Montpellier, INM, INSERM, Département de Neurologie, CRC sclérose en plaques, CRMR LEUKOFRANCE & Centre d’investigation clinique U1411, CHU de Montpellier, Montpellier, France

^4^ Department of Neurology, European Hospital of Marseille, Marseille, France

^5^ Department of Neurology and Clinical Investigation Center, Strasbourg University Hospital, Strasbourg, France

^6^ Department of Neurology, Amiens University Hospital, Amiens, France

^7^ Neurology Unit, GHNE Paris Saclay Hospital, Orsay, France; Université Paris-Saclay, UNIACT, Neurospin – INSERM UMR 1129, CEA, Gif-sur-Yvette, France

^8^ University of Lille, INSERM U1172 LilNCog, Lille University Hospital, FHU Precise, Lille, France

^9^ Department of Neurology, CRC-SEP, Toulouse University Hospital, Toulouse, France; INSERM UMR1291 – CNRS UMR5051, INFINITY, University Toulouse III, Toulouse, France

**Corresponding author:**

Jonathan Ciron

[ciron.j@chu-toulouse.fr](mailto:ciron.j@chu-toulouse.fr)

**Online Resource 2** Round 1 Delphi results used to establish clinical case classification

| **Type of profile** | **Not severe** | **Intermediate** | | | **Severe** | | | |
| --- | --- | --- | --- | --- | --- | --- | --- | --- |
| **Round 1 results: Would you recommend initiating a HET for this patient if presentation upon initial profile?** | | | | | | | | |
| % in favour of no HET | 86% (consensus) | 20% | 17% | 3% | 3% | 0% | 0% | 0% |
| % in favour of HET | 0% | 37% | 67% | 47% | 83% (consensus) | 93% (consensus) | 97% (consensus) | 97% (consensus) |
| **Classification established based on Round 1 results** | **Clinical case 1** | **Clinical case 2** | **Clinical case 3** | **Clinical case 4** | **Clinical case 5** | **Clinical case 6** | **Clinical case 7** | **Clinical case 8** |

*HET* high-efficacy therapy
