# Supplementary material for: DELPHI expert consensus-derived factors influencing the early use of monoclonal high-efficacy therapies in treatment-naïve patients with relapsing-remitting multiple sclerosis
Source: J Neurol. 2026 Oct 22;273(11):679. doi: 10.1007/s00415-026-14167-4 (PMC13690885; doi:10.1007/s00415-026-14167-4)
Supplement: Supplementary file 3 [file 415_2026_14167_MOESM3_ESM.docx]

**DELPHI expert consensus-derived factors influencing the early use of monoclonal high-efficacy therapies in treatment-naïve patients with relapsing-remitting multiple sclerosis**

Journal : Journal of Neurology

Bertrand Bourre^1^, Olivier Guerrier^2^, Xavier Ayrignac^3^, Laurent Suchet^4^, Jérôme de Seze^5^, Ines Masmoudi^6^, Vito AG Ricigliano^7^, Patrick Vermersch^8^, Jonathan Ciron^9^ on behalf of the CAReSEP working group.

^1^ CHU Rouen, Department of Neurology and Inserm CIC-CRB 1404, F-76000, Rouen, France

^2^ Department of Neurology and Neurovascular Unit, Valence Hospital, Valence, France

^3^ Univ Montpellier, INM, INSERM, Département de Neurologie, CRC sclérose en plaques, CRMR LEUKOFRANCE & Centre d’investigation clinique U1411, CHU de Montpellier, Montpellier, France

^4^ Department of Neurology, European Hospital of Marseille, Marseille, France

^5^ Department of Neurology and Clinical Investigation Center, Strasbourg University Hospital, Strasbourg, France

^6^ Department of Neurology, Amiens University Hospital, Amiens, France

^7^ Neurology Unit, GHNE Paris Saclay Hospital, Orsay, France; Université Paris-Saclay, UNIACT, Neurospin – INSERM UMR 1129, CEA, Gif-sur-Yvette, France

^8^ University of Lille, INSERM U1172 LilNCog, Lille University Hospital, FHU Precise, Lille, France

^9^ Department of Neurology, CRC-SEP, Toulouse University Hospital, Toulouse, France; INSERM UMR1291 – CNRS UMR5051, INFINITY, University Toulouse III, Toulouse, France

**Corresponding author:**

Jonathan Ciron

[ciron.j@chu-toulouse.fr](mailto:ciron.j@chu-toulouse.fr)

**Online Resource 3** Results following the Round 1 of the Delphi study conducted in France used to explore expert consensus & identify key factors driving the initiation of monoclonal HET among treatment-naïve relapsing-remitting multiple sclerosis patients

| N. | Question : Would you recommend initiating a monoclonal HET for this patient, if…? | Most frequent answer; consensus level (%) | | | Median |
| --- | --- | --- | --- | --- | --- |
|  |  | Lack of consensus (<80%) | Moderate consensus  (80-90%) | Strong  consensus  (≥90%) |  |
| ***Clinical case 1*** ***–*** *25-year-old Caucasian woman without comorbidities, presenting with an initial attack without motor impairment or ataxia, and without cognitive impairment. Brain MRI shows a low brain lesion load, with no infratentorial lesions, no spinal cord involvement, and 1 Gd-enhancing lesion.* | | | | | |
| Q1 | Presentation upon initial profile |  | No (**86%**) |  | 1 |
| Q2 | Presence of cognitive impairment | No (**40%**) |  |  | 3 |
| Q3 | Presence of a previous attack in the past 12 months | Yes (**76%**) |  |  | 4 |
| Q4 | Presence of motor impairment and/or ataxia | Yes (**60%**) |  |  | 4 |
| Q5 | Presence of a moderate brain lesion load on brain MRI | Yes (**53%**) |  |  | 4 |
| Q6 | Presence of a high brain lesion load on brain MRI |  | Yes (**80%**) |  | 5 |
| Q7 | Presence of 1 infratentorial lesion | Yes (**43%**) |  |  | 3 |
| Q8 | Presence of 2 infratentorial lesions | Yes (**67%**) |  |  | 4 |
| Q9 | Presence of ≥3 infratentorial lesions | Yes (**70%**) |  |  | 5 |
| Q10 | Presence of 1 spinal cord lesion | Yes (**54%**) |  |  | 4 |
| Q11 | Presence of 2 spinal cord lesions |  |  | Yes (**90%**) | 5 |
| Q12 | Presence of ≥3 spinal cord lesions |  |  | Yes (**97%**) | 5 |
| Q13 | Presence of ≥3 Gd-enhancing lesions | Yes (**74%**) |  |  | 4 |
| ***Clinical case 2*** ***–*** *32-year-old Maghrebi man without comorbidities, presenting with an initial attack without motor impairment or ataxia, and without cognitive impairment. Brain MRI shows a low brain lesion load, with no infratentorial lesions, 1 spinal cord lesion, and 1 Gd-enhancing lesion.* | | | | | |
| Q1 | Presentation upon initial profile | Uncertain (**43%**) |  |  | 3 |
| Q2 | The patient is not of Maghrebi origin | No (**53%**) |  |  | 2 |
| Q3 | Presence of cognitive impairment | Yes (**64%**) |  |  | 4 |
| Q4 | Presence of a previous attack in the past 12 months |  | Yes (**86%**) |  | 5 |
| Q5 | Presence of motor impairment and/or ataxia |  |  | Yes (**90%**) | 4 |
| Q6 | Presence of a moderate brain lesion load on brain MRI |  | Yes (**83%**) |  | 4 |
| Q7 | Presence of a high brain lesion load on brain MRI |  |  | Yes (**96%**) | 5 |
| Q8 | Presence of 1 infratentorial lesion |  | Yes (**80%**) |  | 4 |
| Q9 | Presence of 2 infratentorial lesions |  | Yes (**86%**) |  | 5 |
| Q10 | Presence of ≥3 infratentorial lesions |  |  | Yes (**90%**) | 5 |
| Q11 | Absence of spinal cord lesions | No (**70%**) |  |  | 2 |
| Q12 | Presence of 2 spinal cord lesions |  |  | Yes (**90%**) | 5 |
| Q13 | Presence of ≥3 spinal cord lesions |  |  | Yes (**97%**) | 5 |
| Q14 | Presence of ≥3 Gd-enhancing lesions |  | Yes (**86%**) |  | 5 |
| ***Clinical case 3*** ***–*** *27-year-old Caucasian man without comorbidities, presenting with an initial attack with motor impairment and/or ataxia, and without cognitive impairment. Brain MRI shows a moderate brain lesion load, with 1 infratentorial lesion, no spinal cord involvement, and 1 Gd-enhancing lesion.* | | | | | |
| Q1 | Presentation upon initial profile | Yes (**67%**) |  |  | 4 |
| Q2 | Presence of cognitive impairment | Yes (**77%**) |  |  | 4 |
| Q3 | Presence of a previous attack in the past 12 months |  | Yes (**87%**) |  | 5 |
| Q4 | Absence of motor impairment and/or ataxia | No (**47%**) |  |  | 3 |
| Q5 | Presence of a low brain lesion load on brain MRI | No (**40%**) |  |  | 3 |
| Q6 | Presence of a high brain lesion load on brain MRI |  |  | Yes (**90%**) | 5 |
| Q7 | Absence of infratentorial lesions | No (**40%**) |  |  | 3 |
| Q8 | Presence of 2 infratentorial lesions | Yes (**77%**) |  |  | 4 |
| Q9 | Presence of ≥3 infratentorial lesions |  | Yes (**87%**) |  | 5 |
| Q10 | Presence of 1 spinal cord lesion |  | Yes (**86%**) |  | 4 |
| Q11 | Presence of 2 spinal cord lesions |  |  | Yes (**93%**) | 5 |
| Q12 | Presence of ≥3 spinal cord lesions |  |  | Yes (**100%**) | 5 |
| Q13 | Presence of ≥3 Gd-enhancing lesions |  |  | Yes (**94%**) | 5 |
| ***Clinical case 4*** *– 35-year-old Afro-Caribbean woman without comorbidities, presenting with an initial attack without motor impairment or ataxia, with cognitive impairment. Brain MRI shows a low brain lesion load, with no infratentorial lesions, no spinal cord involvement, and 3 Gd-enhancing lesions.* | | | | | |
| Q1 | Presentation upon initial profile | Uncertain (**50%**) |  |  | 3 |
| Q2 | The patient is not of Afro-Caribbean origin | Uncertain (**43%**) |  |  | 3 |
| Q3 | Absence of cognitive impairment | Uncertain (**40%**) |  |  | 3 |
| Q4 | Presence of a previous attack in the past 12 months |  |  | Yes (**90%**) | 5 |
| Q5 | Presence of motor impairment and/or ataxia |  | Yes (**87%**) |  | 5 |
| Q6 | Presence of a moderate brain lesion load on brain MRI |  |  | Yes (**90%**) | 4 |
| Q7 | Presence of a high brain lesion load on brain MRI |  |  | Yes (**97%**) | 5 |
| Q8 | Presence of 1 infratentorial lesion | Yes (**73%**) |  |  | 4 |
| Q9 | Presence of 2 infratentorial lesions |  |  | Yes (**90%**) | 5 |
| Q10 | Presence of ≥3 infratentorial lesion |  |  | Yes (**100%**) | 5 |
| Q11 | Presence of 1 spinal cord lesion |  |  | Yes (**94%**) | 4 |
| Q12 | Presence of 2 spinal cord lesions |  |  | Yes (**96%**) | 5 |
| Q13 | Presence of ≥3 spinal cord lesions |  |  | Yes (**100%**) | 5 |
| Q14 | Presence of 0-1-2 Gd-enhancing lesion(s) | No (**57%**) |  |  | 2 |
| ***Clinical case 5*** *– 52-year-old Caucasian man without comorbidities, presenting with an initial attack without clinically concerning symptoms, and without motor impairment or ataxia, with cognitive impairment. Brain MRI shows a high brain lesion load, with 3 infratentorial lesions, 1 spinal cord lesion, and 1 Gd-enhancing lesion.* | | | | | |
| Q1 | Presentation upon initial profile |  | Yes (**83%**) |  | 5 |
| Q2 | Absence of cognitive impairment |  | Yes (**83%**) |  | 5 |
| Q3 | Presence of a previous attack in the past 12 months |  |  | Yes (**97%**) | 5 |
| Q4 | Presence of motor impairment and/or ataxia |  |  | Yes (**93%**) | 5 |
| Q5 | Presence of a low brain lesion load on brain MRI | Yes (**62%**) |  |  | 4 |
| Q6 | Presence of a moderate brain lesion load on brain MRI | Yes (**79%**) |  |  | 5 |
| Q7 | Absence of infratentorial lesions | Yes (**79%**) |  |  | 4 |
| Q8 | Presence of 1 infratentorial lesion | Yes (**79%**) |  |  | 4 |
| Q9 | Presence of 2 infratentorial lesions |  | Yes (**83%**) |  | 5 |
| Q10 | Absence of spinal cord lesions | Yes (**75%**) |  |  | 4 |
| Q11 | Presence of 2 spinal cord lesions |  |  | Yes (**93%**) | 5 |
| Q12 | Presence of ≥3 spinal cord lesions |  |  | Yes (**100%**) | 5 |
| Q13 | Presence of ≥3 Gd-enhancing lesions |  |  | Yes (**100%**) | 5 |
| ***Clinical case 6*** *– 55-year-old Caucasian woman with COPD, presenting an initial attack with motor impairment and/or ataxia, without cognitive impairment. Brain MRI shows a moderate brain lesion load, with no infratentorial lesions, 2 spinal cord lesions, and 2 Gd-enhancing lesions.* | | | | | |
| Q1 | Presentation upon initial profile |  |  | Yes (**93%**) | 5 |
| Q2 | Absence of comorbidities |  |  | Yes (**96%**) | 5 |
| Q3 | Presence of cognitive impairment |  |  | Yes (**97%**) | 5 |
| Q4 | Presence of a previous attack in the past 12 months |  |  | Yes (**100%**) | 5 |
| Q5 | Absence of motor impairment and/or ataxia | Yes (**73%**) |  |  | 5 |
| Q6 | Presence of a low brain lesion load on brain MRI |  | Yes (**83%**) |  | 5 |
| Q7 | Presence of a high brain lesion load on brain MRI |  |  | Yes (**96%**) | 5 |
| Q8 | Presence of 1 infratentorial lesion |  |  | Yes (**97%**) | 5 |
| Q9 | Presence of 2 infratentorial lesions |  |  | Yes (**97%**) | 5 |
| Q10 | Presence of ≥3 infratentorial lesions |  |  | Yes (**100%**) | 5 |
| Q11 | Absence of spinal cord lesions | Uncertain (**50%**) |  |  | 3 |
| Q12 | Presence of 1 spinal cord lesion |  |  | Yes (**97%**) | 5 |
| Q13 | Presence of ≥3 spinal cord lesions |  |  | Yes (**100%**) | 5 |
| Q14 | Presence of ≥3 Gd-enhancing lesions |  |  | Yes (**100%**) | 5 |
| ***Clinical case 7*** *–* *30-year-old Caucasian woman with cardiovascular comorbidities, presenting with an initial attack without motor impairment or ataxia, with cognitive impairment. Brain MRI shows a moderate brain lesion load with 1 infratentorial lesion, no spinal cord involvement, and 4 Gd-enhancing lesions.* | | | | | |
| Q1 | Presentation upon initial profile |  |  | Yes (**97%**) | 5 |
| Q2 | Absence of comorbidities |  |  | Yes (**97%**) | 5 |
| Q3 | Absence of cognitive impairment | Yes (**63%**) |  |  | 4 |
| Q4 | Presence of a previous attack in the past 12 months |  |  | Yes (**100%**) | 5 |
| Q5 | Presence of motor impairment and/or ataxia |  |  | Yes (**100%**) | 5 |
| Q6 | Presence of a low brain lesion load on brain MRI | Yes (**73%**) |  |  | 4 |
| Q7 | Presence of a high brain lesion load on brain MRI |  |  | Yes (**100%**) | 5 |
| Q8 | Absence of infratentorial lesions |  | Yes (**86%**) |  | 4 |
| Q9 | Presence of 2 infratentorial lesions |  |  | Yes (**100%**) | 5 |
| Q10 | Presence of ≥3 infratentorial lesions |  |  | Yes (**100%**) | 5 |
| Q11 | Presence of 1 spinal cord lesion |  |  | Yes (**100%**) | 5 |
| Q12 | Presence of 2 spinal cord lesion |  |  | Yes (**100%**) | 5 |
| Q13 | Presence of ≥3 spinal cord lesions |  |  | Yes (**100%**) | 5 |
| Q14 | Presence of 0-1-2 Gd-enhancing lesion(s) | Yes (**50%**) |  |  | 4 |
| ***Clinical case 8*** *– 30-year-old Caucasian woman without comorbidities, presenting with an initial attack with motor impairment and/or ataxia, without cognitive impairment. Brain MRI shows a high brain lesion load, with 2 infratentorial lesions, 6 spinal cord lesions, and 1 Gd-enhancing lesion.* | | | | | |
| Q1 | Presentation upon initial profile |  |  | Yes (**97%**) | 5 |
| Q2 | Presence of cognitive impairment |  |  | Yes (**100%**) | 5 |
| Q3 | Presence of a previous attack in the past 12 months |  |  | Yes (**100%**) | 5 |
| Q4 | Absence of motor impairment and/or ataxia |  |  | Yes (**96%**) | 5 |
| Q5 | Presence of a low brain lesion load on brain MRI |  |  | Yes (**97%**) | 5 |
| Q6 | Presence of a moderate brain lesion load on brain MRI |  |  | Yes (**96%**) | 5 |
| Q7 | Absence of infratentorial lesions |  |  | Yes (**97%**) | 5 |
| Q8 | Presence of 1 infratentorial lesion |  |  | Yes (**96%**) | 5 |
| Q9 | Presence of ≥3 infratentorial lesions |  |  | Yes (**100%**) | 5 |
| Q10 | Absence of spinal cord lesions |  |  | Yes (**93%**) | 5 |
| Q11 | Presence of 1 spinal cord lesion |  |  | Yes (**94%**) | 5 |
| Q12 | Presence of 2 spinal cord lesions |  |  | Yes (**97%**) | 5 |
| Q13 | Presence of ≥3 Gd-enhancing lesions |  |  | Yes (**100%**) | 5 |

*COPD* chronic obstructive pulmonary disease*; Gd* gadolinium*; HET* high-efficacy therapy*; MRI* magnetic resonance imaging
